# Single Cell MicroRNA Analysis Using Microfluidic Flow Cytometry

**DOI:** 10.1371/journal.pone.0055044

**Published:** 2013-01-30

**Authors:** Meiye Wu, Matthew Piccini, Chung-Yan Koh, Kit S. Lam, Anup K. Singh

**Affiliations:** 1 Department of Biotechnology and Bioengineering, Sandia National Laboratory, Livermore, California, United States of America; 2 Biochemistry and Molecular Biology Graduate Group, University of California Davis, Davis, California, United States of America; 3 Department of Hematology and Oncology, University of California Davis Medical Center, Sacramento, California, United States of America; Northeastern University, United States of America

## Abstract

MicroRNAs (miRNAs) are non-coding small RNAs that have cell type and cell context-dependent expression and function. To study miRNAs at single-cell resolution, we have developed a novel microfluidic approach, where flow fluorescent *in situ* hybridization (flow-FISH) using locked-nucleic acid probes is combined with rolling circle amplification to detect the presence and localization of miRNA. Furthermore, our flow cytometry approach allows analysis of gene-products potentially targeted by miRNA together with the miRNA in the same cells. We demonstrate simultaneous measurement of miR155 and CD69 in 12-O-tetradecanoylphorbol 13-acetate (PMA) and Ionomycin stimulated Jurkat cells. The flow-FISH method can be completed in ∼10 h, utilizes only 170 nL of reagent per experimental condition, and is the first to directly detect miRNA in single cells using flow cytometry.

## Introduction

miRNAs are non-coding, small single-stranded RNAs that regulate gene expression in numerous biological systems ranging from cell development and differentiation [1], in immune response and inflammation [2], [3], and in pathological states such as cancer [4] and autoimmune diseases [5]. miRNAs function by directly binding the 3′ untranslated regions (UTRs) of target mRNAs and recruit the RNA-induced silencing complex to degrade target mRNA [6]. In humans, over 1000 miRNAs have been identified [7], and each miRNA can potentially repress hundreds of target mRNAs, indicating the importance and complexity of this gene regulation system. Several methods have been developed for detection of miRNAs: Northern blotting [8], oligonucleotide microarrays [9], [10], quantitative PCR assays [11], [12], next generation sequencing [13], and *in situ* hybridization (ISH) [14]. With the exception of qPCR and ISH, all of these methods require lysis and homogenization of cells in order to provide measurement of miRNA averaged over a large number of cells. Cells are heterogeneous in nature and hence, in many applications it is desirable to measure miRNA in single cells. The advent of locked nucleic acid (LNA) containing probes has enabled RT-qPCR [15], [16] and *in situ* hybridization (ISH) analysis [17], [18] of miRNA at single cell resolution. Single cell miRNA RT-qPCR however, requires many steps including isolation of single cells followed by lysis, RNA extraction and amplification, and has limited throughput. LNA-ISH allows detection of endogenous miRNA in single cells without lysis and RNA extraction, but is labor-intensive, at times poorly reproducible and provide qualitative assessment, and hence, is used most frequently for fixed tissue sections. Microfluidic devices have attracted significant attention in single cell analysis [19]–[21]. Leveraging our group’s previous work in developing microfluidic single cell analysis systems including microfluidic bacterial rRNA flow-FISH [22] and cell signaling pathway profiling [23], we report on a novel 10-chamber microfluidic chip platform for multiplexed detection of miRNA and proteins in single cells under 10 different experimental conditions simultaneously. The approach relies on a novel flow-cytometry approach using LNA fluorescent *in situ* hybridization followed by rolling-circle amplification for miRNA detection. Instead of using tyramide signal amplification to visualize the miRNA-LNA probe duplex, we used rolling circle amplification (RCA) of the target miRNA signal to achieve robust and reliable signal amplification. The RCA amplification reagent has been previously used to detect proteins both in lysates [24] and in cells [25], and has a limit of detection between 10^−14^ to 10^−13^ molar [26], providing the necessary sensitivity for detection of miRNA in single cells. An added benefit of LNA-Flow FISH is the possibility of multiplexing with protein immunostaining in the same cell. In this report, we multiplexed miRNA detection with immunostaining of a protein to show the multiplexing capability.

We demonstrate our method in Jurkat cells, a model cell line for the study of T cell activation [27]. PMA and Ionomycin trigger T cell activation, which leads to production of transmembrane glycoprotein CD69 [28] and up-regulation of miR155 [29]. CD69 is a lectin C-type protein that is involved in T cell differentiation through the Jak3/Stat5 pathway, and is the earliest inducible surface protein indicative of T cell activation [30]. miR155 up-regulation in T cells is implicated in the negative regulation of SOCS1 protein, which leads to increased levels of interleukin-2, a cytokine necessary for T cell proliferation [29]. We visualized and quantified PMA and Ionomycin induced CD69 and miR155 in Jurkat cells using both microscopy and flow cytometry, demonstrating simultaneous detection of miRNA and a target protein in the same cell.

## Materials and Methods

### Cell Culture and Stimulation

Jurkat cells were purchased from ATCC (TIB-152), and cultured in RPMI media (11875-093, Invitrogen) containing 10% FBS (100–500, Gemini) and 0.5 mg/ml penicillin and streptomycin (15240062, Invitrogen). For stimulation of Jurkat cells, cells were seeded at 1×10^6^/ml for 0, 8, 16, 20, or 24 h with 10 ng/mL PMA (P8139, Sigma) and 1 µM Ionomycin (I3909, Sigma). After stimulation, 5×10^5^ cells from each condition were set aside for RNA extraction and RT-qPCR, the rest of the cells were fixed with 8% paraformaldehyde (Electron Microscopy Sciences) in PBS for 10 min. Fixed cells were pelleted at 300 g for 5 min, and washed twice with PBS.

### RT- qPCR

5×10^5^ cells from each condition were set aside prior to fixation and pelleted at 300 g for 5 min and washed 2× with PBS. Total RNA was extracted using the RNeasy kit from Qiagen according to manufacturer’s instructions. The extracted RNA was quantified using a Nanodrop 2000, and 100 ng of RNA was used to generate cDNA using the miScript II RT kit (Qiagen). All steps were performed according to manufacturer’s instructions. 100 ng of cDNA from each condition was subsequently used in miScript hsa-mir-155 primer assay (Sanger accession: MI0000681, Qiagen), normalized to positive control SNORD61 (Qiagen). Expression level of miRNA155 was analyzed using the 2^−ΔΔCt^ method. SNORD61 served as positive control for normalization. miR155 level at 0 h served as basal level, and miR155 in PMA and Ionomycin treated samples are expressed as fold changes compared with 0 h.

### Microfluidic Chip Design and Platform Setup

The ten-chamber microfluidic chip was designed in-house using AutoCAD 2010 (Autodesk Inc., San Rafael, CA), photomasks were generated at Photo Sciences (Torrence, CA), and quartz microfluidic devices were fabricated by Caliper Life Sciences (Hopkinton, MA). An array of fourteen holes 500 *µ*m in diameter, seven on each side, provided for fluid inlet. All subsequent steps in chip packaging and details of the chip platform are as previously described [23]. Detailed photographs of the platform are shown in figure S1.

### Microchip Surface Treatment with Cell-Tak™

The planar microfluidic cell preparation chip in this study contains ten fluidically-isolatable chambers with channel dimensions of 200 µm (width), 30 µm depth, 72 mm length, each holding up to 2000 macrophages and 170 nL of fluid volume. The 10-channel microchip was cleaned with 10% bleach in filtered DI water for 15 min, followed by flushing with DI to wash off all residual bleach. The working Cell-Tak™ (354240, BD Biosciences) solution (15 µl Cell-Tak™, 575 µl 0.1 M sodium bicarbonate pH 8.0, and 10 µl of 1N NaOH) was introduced into the chip for at least 15 min, followed by PBS flush for 5 min. Fixed Jurkat cells were introduced into the Cell-Tak™ coated chip and captured on the microchannel surface for ISH.

### On-chip LNA *in situ* Hybridization

The miR155 (/5DigN/ACCCCTATCACGATTAGCATTAA/3Dig_N/) and scrambled (/5DigN/GTGTAACACGTCTATACGCCCA/3DIG_N/) LNA double DIG-labeled probes were purchased from Exiqon. Fixed Jurkat cells were loaded into the chip, and allowed to settle and adhere to the micro channel surface for 30 min. During the settling time, the following solutions were made fresh: solution 1 (0.13 M 1-methylimidazole, 300 mM NaCl, pH 8.0, adjusted with HCl), EDC solution (0.16 M EDC in solution 1, adjusted to pH 8.0), hybridization buffer (50% formamide, 2×SSC, 50 µg/ml yeast tRNA, 50 µg/ml salmon sperm DNA, 50 mM NaPi). To permeabilize the Jurkat cells, 0.25% Triton-X 100 in TBS was flown into all chambers for 10 min, followed by 5 min wash with solution 1. After incubation with solution 1, solution 2 was flown into all chambers and cells were incubated for 20 min at RT, followed by a 5 min wash with TBS. The cells were then pre-hybridized for 30 min at 62°C in hybridization buffer pre-warmed to 65°C. All LNA probes were used at 10 pmol/25 µl hybridization buffer. The hybridization with LNA probes were performed at 80°C for 90s, followed by 90 min at 62°C. Following LNA probe hybridization, all chambers were washed with 2×SSC +50% Formamide at 65°C for 10 min (flow 5 min, stop 5 min), then washed with 1×SSC for 20 min (flow 5 min, stop 15 min) at RT, and finally washed with 0.1×SSC for 20 min (flow 5 min, stop 15 min) at RT.

### Signal Amplification Using Rolling Circle Amplification

To amplify the LNA probe bound miRNA signals, the FITC Duolink mouse PLUS (92001-0030) and mouse MINUS (92004-0030) probes and green detection kit (92014-0030) from Olink Biosciences were used to perform rolling circle amplification of miRNA signals. After *in situ* hybridization, the cells were blocked with 2% BSA for 30 min at 37°C, followed by incubation with anti-DIG antibody (11333062910, Roche) at 1∶50 for 1 h at 37°C. Cells were then washed with TBST for 5 min. The Duolink mouse PLUS and MINUS probes were diluted at 1∶5 (20 µl PLUS +20 µl MINUS +60 µl dilution buffer from kit), flow into all chambers, and incubated for 1 h at 37°C. After probe incubation, all chambers were washed for 5 min with TBST. The ligation and amplification steps were done according to manufacturer’s instructions, using only 1 reaction volume for all 10 chambers. After on-chip sample preparation, cells were detached via proteolytic cleavage using 100 µg/mL elastase (I.U.B.: 3.4.21.36, Worthington), and hydrodynamically focused for on-chip flow cytometry.

### CD69 Protein Immunostaining Multiplexed with LNA Flow-FISH

To multiplex protein immunostaining with LNA flow-FISH, Jurkat cells were stained with anti-CD69-biotin antibody at 1∶100 (13-0699-80, eBioscience) in PBS for 30 min at RT prior to permeabilization with 0.25% Triton. A solution of CD69 antibody was flown into all chambers, flow was then stopped for 30 min for incubation. All chambers were washed with TBS with 0.05% Tween for 5 min, followed by 30 min incubation with a 1 nM solution of Qdot 705 streptavidin conjugate (Q10161MP, Invitrogen) in PBS. Following Qdot 705 Streptavidin incubation, all chambers were washed with TBST for 5 min. All chambers were then washed with TBST for 5 min. The *in situ* hybridization procedure continues from this point on at the permeabilization step.

### Microscopy and Image Analysis

Prior to imaging, the cells were incubated with Hoechst stain (33342, Pierce) in PBS for 10 min, followed by 10 min wash in PBS. Epi-fluorescent images were captured at 60X magnification on an Olympus IX-71 microscope equipped with GFP, Texas Red, DAPI filters and Hamamatsu ORCA-R2 cooled CCD camera controlled via free micro-manager software. Images were artificially colored and overlaid in ImageJ.

### On-chip Laser Induced Fluorescence and Flow Cytometry

A 20-mW diode pumped solid-state laser at 488-nm (85-BCF-020-112; CVI Melles Griot, Carlsbad, CA), in an epi-fluorescence configuration was used for excitation. The laser beam was reflected and focused upon the detection region using a long pass dichroic mirror (LPD01-488S; Semrock, Rochester, NY) and an aspheric lens (5722-H-B; New Focus, Santa Clara, CA), respectively. Forward scattering was collected and channeled to the active area of a photomultiplier tube (PMT) based detector (H5784-20; Hamamatsu, Bridgewater, NJ) using a custom made sculpted tip silica optical fiber (1000 *µ*m core, 2000 µm sculpted spherical tip; Polymicro Technologies, Phoenix, AZ). Laser-induced fluorescence emission was first collected via the same aspheric lens used for focusing and subsequently passed on for detection via the dichroic mirror used in the excitation leg of the apparatus as described above. For multiplexed detection an eight channel Hamamatsu linear multi-anode (LMA) PMT coupled with filter optics (H9797TM; Hamamatsu, Bridgewater, NJ) was used. Only four channels of the LMA PMT were used in this configuration, and the filtering was selected for green, yellow, red, and far-red fluorescence detection. The green fluorescence was detected using longpass dichroic mirror (DMT560: Hamamatsu, Bridgewater, NJ) and a bandpass filter (BPF534_30). For yellow fluorescence detection in the second channel, longpass dichroic mirror (DMT650) and bandpass filter (BPF585_40). Red fluorescence was detected third channel of the LMA PMT via a longpass dichroic (DMT740) and filtered using a third bandpass filter (BPF692_40). Finally, far-red fluorescence detection was accomplished by cascading the remaining florescence signal onto the fourth LMA PMT channel using a mirror, and filtered onto that channels detection region using a fourth bandpass filter (BPF785_62). Data acquisition was performed for all five photomultiplier voltages (488-nm scatter, green, yellow, red and far-red) by a data acquisition module (NI USB-6259; National Instruments, Austin, TX). In-house software for data acquisition and recording was scripted using LabView, the data was further analyzed and processed using a custom “Peak Finder” application also scripted using LabVIEW.

## Results and Discussion

### Microfluidic Platform and Assay Design

**Figure 1:**
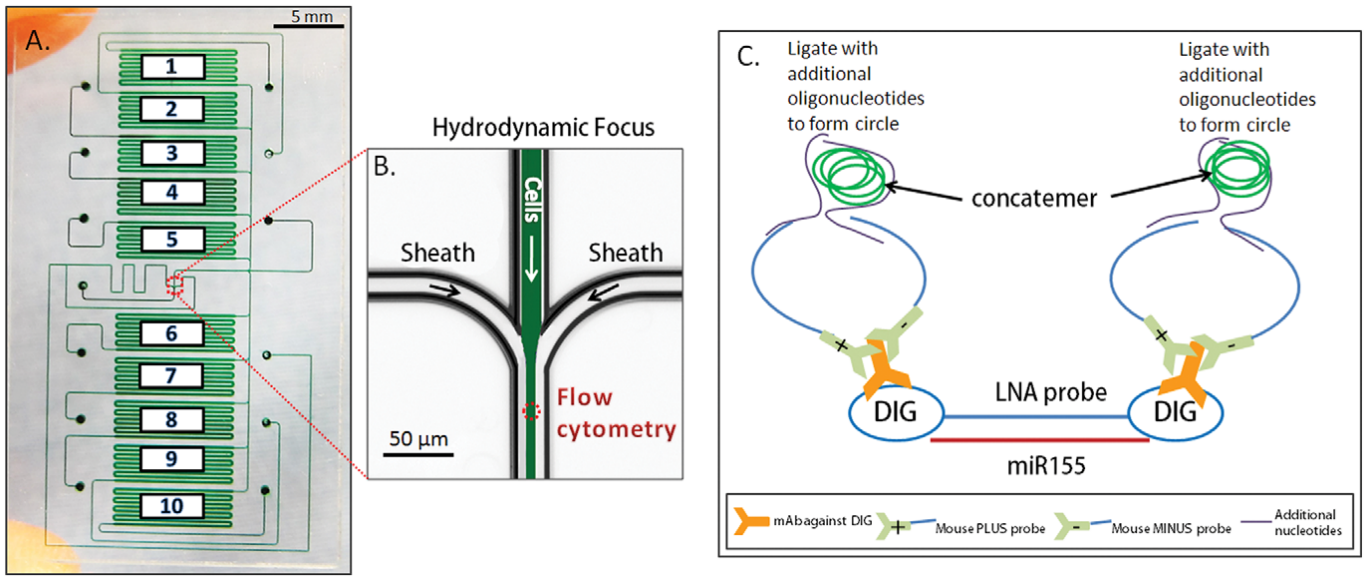
Microfluidic chip and LNA flow-FISH method schematic. A. 10-chamber microfluidic chip. Cells prepared in each of the holding chambers can be detached and driven to the center of the chip shown in B, for hydrodynamic focus and flow cytometry. The signal from miRNA155 hybridized to the LNA probe is amplified using RCA method depicted in C. The DIG label on both ends of the LNA probe is recognized by anti-DIG mAb, and two RCA probes (+ and -) bind to each anti-DIG mAb and are ligated to two additional oligonucleotides to form circular templates for rolling circle amplification. The resultant concatemer products can be visualized using microscopy as well as measured using flow cytometry.

**Figure 2:**
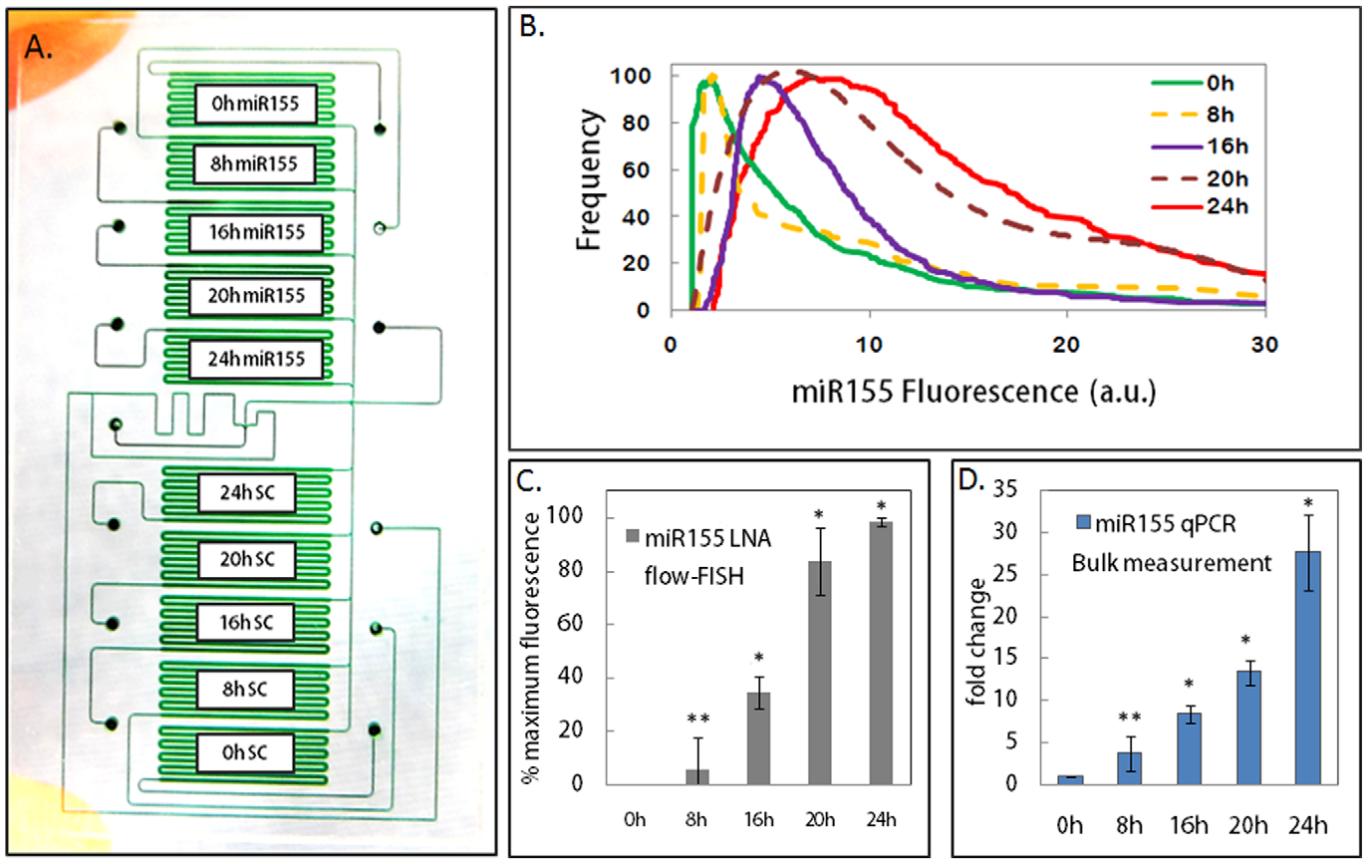
LNA Flow-FISH detection of miR155 in Jurkat cells activated by PMA and Ionomycin. Jurkat cells stimulated with PMA and Ionomycin for 0, 8, 16, 20, and 24 h were loaded in duplicate into the 10-chamber chip as shown in A. The top 5 chambers were hybridized with miR155 probe, whereas the bottom 5 chambers were hybridized to scrambled probe for negative control. B. overlay of miR155 fluorescence histograms measured by LNA flow-FISH. C. miR155 median fluorescence normalized as a percentage of the maximum fluorescence, showing significant increase from 0 h at 16, 20, and 24 h stimulation. (* p<0.01) The increase at 8 h was not statistically significant (** p>0.01). D. RT-qPCR of miR155 fold changes in Jurkat cells treated with PMA and Ionomycin, also showing significant miR155 increase starting at 16 h stimulation, but not at 8 h. (** p>0.01, * p<0.01).

A 10-chamber microfluidic chip was designed for sample preparation and on-chip flow cytometry shown in figure 1A. The chip has fluidically-isolatable chambers, each capable of holding up to 2000 cells. The microchannel surfaces are pre-coated with Cell Tak™ solution to facilitate capture of non-adherent Jurkat cells for the on-chip hybridization and immunostaining. After sample preparation, the cells in the chambers are detached using proteolytic cleavage, and driven by positive pressure to the center of the chip where they are hydrodynamically focused (figure 1B) and interrogated using micro-flow cytometry using a custom built setup (figure S1C). Our microfluidic platform’s ultra low reagent consumption substantially reduces the cost of LNA flow-FISH (∼100-fold reduction from ∼$150/sample to <$1.50/sample). The flow-FISH assay uses a novel combination of LNA probes with RCA signal amplification for a robust miRNA signal that can be quantified by flow cytometry as well as visualized by microscopy. A schematic depicting the miRNA LNA Flow-FISH method is shown in figure 1C. Once the cells are loaded into the chambers and captured by Cell Tak™, miR155 LNA probe with digoxigenin (DIG) conjugated to both 5′ and 3′ ends is hybridized to mature intracellular miR155. After the LNA probe hybridizes with miR155, monoclonal anti-DIG antibodies (mAb) bind the DIG labels at both ends of the LNA probe. A pair of antibody/oligonucleotide probes (one positive, one negative) are used to bind to the anti-DIG antibodies. After binding to the anti-DIG antibody, the two antibody/oligonucleotide probes are ligated with two additional oligonucleotides to form a circular template for the subsequent rolling circle amplification with Phi29 bacterial polymerase. FITC-labeled oligonucleotide detection probes complementary to the ligated circular template are hybridized with the resultant circular concatemers, and each RCA amplified LNA-probe/miRNA duplex becomes visible as fluorescent dots (figure 2A), and can also be detected by flow cytometry. RCA amplification provides improved signal specificity because it occurs only when a circular template is generated, and the detection of the circular product is accomplished using sequence-specific hybridization probes.

### LNA Flow-FISH Analysis of PMA and Ionomycin Induced miR155 Upregulation

To track the expression of PMA and Ionomycin induced miR155 over time, Jurkat cells stimulated with PMA and Ionomycin for 0, 8, 16, 20, and 24 h were loaded in duplicate into the 10 chamber chip (figure 2A). The top 5 chambers were hybridized with double DIG labeled miR155 LNA probe, and the bottom 5 chambers were hybridized with double DIG labeled random scrambled probe as negative control. The RCA amplified miR155 fluorescence was quantified using on-chip flow cytometry and the fluorescence histograms are overlaid and shown in figure 2B. The normalized median fluorescence values from three separate experiments were plotted as percent of maximal fluorescence from each sample in figure 2C. Both the overlay in 2B and the bar graph of median values in 2C show incremental increase in miR155 for Jurkat cells under stimulation with PMA and Ionomycin. Hybridization to the random scrambled probe produced low level of background fluorescence that remained constant throughout the time course, and the background was subtracted from the miR155 measurements. The miRNA flow-FISH results were verified using population RT-qPCR analysis and calculated relative fold change from 0 h (figure 2D). Both flow-FISH and qPCR showed that miR155 increase significantly from 0 h at 16 h, 20 h, and 24 h, with *p* values <0.01; miR155 increased from 0 to 8 h, but the change was not statistically significant (*p*>0.1). The upregulation of miR155 by PMA and Ionomycin detected by our LNA flow-FISH method corroborates the existing findings of miR155 upregulation in activated T cells and not in resting T cells [31].

### Multiplexed Analysis of CD69 Protein Expression and miR155 Upregulation

**Figure 3:**
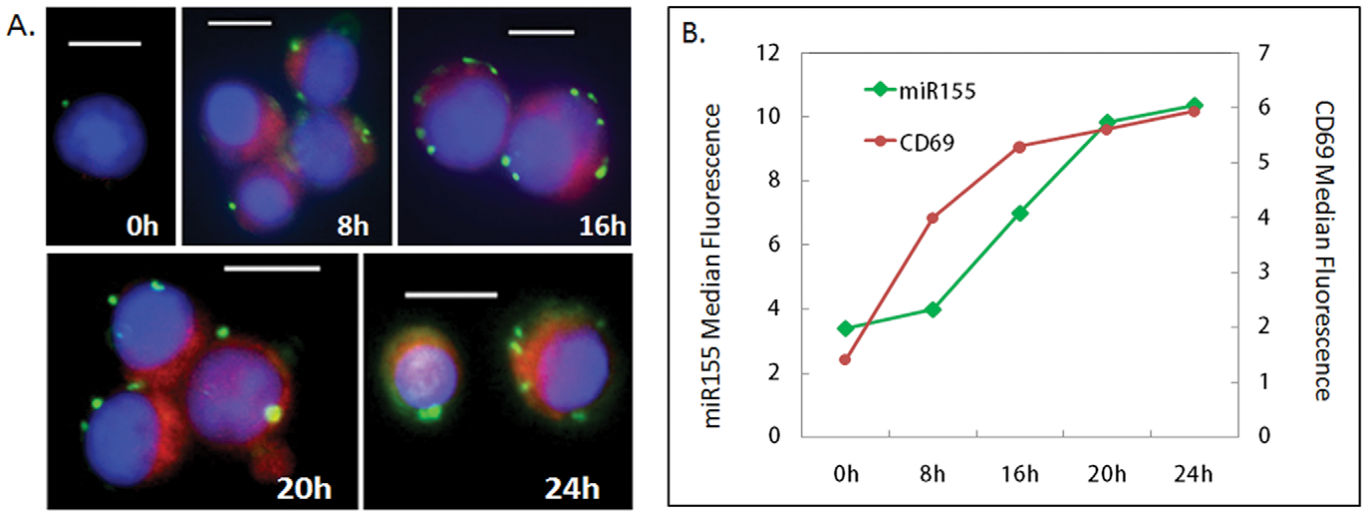
Multiplexed detection of CD69 and miR155 using imaging and flow-FISH. A. Images of Jurkat cells showing RCA amplified miR155 signal as green dots in the cytosol, CD69 proteins stained with Qdot705 as red, and the nucleus is in blue. B. Median values of multiplexed miR155 fluorescence and CD69-Qdot705 fluorescence collected via flow cytometry.

The high melting temperature of LNA probes provides superior specificity and rapid hybridization for detecting small miRNAs, but the high hybridization temperature can reverse formaldehyde fixation, and miRNAs can be washed away. A second fixation step (figure S2) using 1-ethyl-3-(3-dimethyl-aminopropyl) carbodiimide or EDC was performed subsequent to formaldehyde fixation to irreversibly cross-link the miRNA to the neighboring amino acid residues [17]. The EDC fixation step retains miRNAs inside the cell, but destroys protein epitopes and protein based fluorephores such as phycoerytherin. To solve this problem, we used quantum dot labeled secondary antibody for multiplexed protein detection with LNA flow-FISH because quantum dots are inert to EDC fixation. Immunostaining with anti-CD69 antibody and Qdot 705 labeled secondary antibody was performed prior to fixation by EDC. At time 0, no CD69 and almost no miR155 can be seen. The size and intensity of green miR155 dots increase as duration of stimulation increases. After microscopy, the cells were detached and analyzed using flow cytometry, the median fluorescence values from miR155 and CD69 are plotted in the same graph (figure 3B), showing increase of both miR155 and CD69 under PMA and Ionomycin stimulation. Significant CD69 induction can be detected 8 h post stimulation. miR155 upregulation occurred later than CD69 protein induction, detectable starting at 16 h, indicating that signaling events surrounding CD69 protein induction was activated earlier by PMA and Ionomycin than those that lead to miR155 upregulation.

### Conclusion

We have developed a novel flow-FISH method for measuring relative miRNA changes at single cell resolution using a 10-chamber microfluidic chip platform. The biggest advantage of the flow-FISH method is the capability to multiplex the detection of miRNAs with protein immunostaining in the same cell, and preserve the cell-to-cell heterogeneity within the population (Figure S3). While we have shown detection of a single protein, it is possible to detect multiple proteins in the same cell using antibodies labeled with different fluorephores or quantum dots. This multiplexing capability opens up many potential applications for miRNA flow-FISH in both clinical and basic biological sciences. One potential clinical application is the profiling of miRNA levels in complex clinical samples such as peripheral blood mononuclear cells (PBMCs) from patients with leukemia or autoimmune disorders. The expression level of up to 10 different miRNA in 10 immune subsets can be analyzed in one microfluidic chip experiment. miRNA expression levels in many different cell types can be assessed and tracked as biomarkers indicative of disease progression or response to therapy. If miRNA copy number quantitation is required for development of a specific clinical diagnostic assay, custom flow-FISH quantitation beads can be developed by conjugating known numbers of synthetic miRNAs onto polystyrene beads. The calibration beads can be captured on the chip and hybridized to the same LNA FISH probe and amplified on the chip along with experimental samples under the same conditions to provide a standard curve from which the miRNA copy number per cell can be determined.

For application in basic science, miRNA flow-FISH can be used to develop functional assays for the discovery of miRNA targets *in vivo*. One miRNA and dozens of its putative mRNA and protein targets can be simultaneously quantified using a combination of LNA flow-FISH for miRNAs, traditional flow-FISH for the mRNAs, and immunostaining for the proteins. Time course experiments tracking the miRNA and target mRNA/protein levels in the presence of miRNA antagonists or mimics can validate mRNA and protein targets *in vivo*. Finally, since we have already successfully demonstrated the spatiotemporal profiling of protein signaling pathway using a microfluidic platform [23], combining miRNA flow-FISH with cell signaling pathway profiling will provide a comprehensive look into the relationship between miRNAs and the cell signaling pathways they modulate.

## Supporting Information

Figure S1 **Details of the microfluidic platform.** The planar microfluidic chip sits in a manifold designed in-house (A). Tubing connect valves and reagent reservoires to the inlets on the microfluid chip. B. The manifold is retrofitted to a commercial Olympus IX71 microscope. In-house designed software allows the experimenter to control the pressure, temperature, and valves by programming each step of the experiment to run automatically. After sample preparation, the manifold is moved to the micro flow cytometer setup shown in C, and on-chip flow cytometry is performed. The optical fiber is positioned on top of the chip, and aligned to the hydrodynamically focused path of the cells. The laser is applied from the bottom of the chip, and the signal from the the passing cells are recorded by the PMTs situlated underneath the chip.
(PDF)

Figure S2 **Schematic of EDC fixation mechanism.** The 5′ phosphate of miRNA are activated by EDC to form an intermediate that is crosslinked to neighboring amino groups of proteins. The fixation with EDC allows irreversible crosslinking to tether miRNAs inside the cells during the high temperature hybridization with LNA containing probes.
(PDF)

Figure S3 **Scatter plot of Jurkat cells after 24 h of PMA and Ionomycin activation, showing heterogeneity of CD69 and miR155 expression levels in individual cells.**
(TIF)
