# Supplementary figures and images for: Single Cell MicroRNA Analysis Using Microfluidic Flow Cytometry
Source: PLoS One. 2013 Jan 30;8(1):e55044. doi: 10.1371/journal.pone.0055044 (PMC3559333; doi:10.1371/journal.pone.0055044)

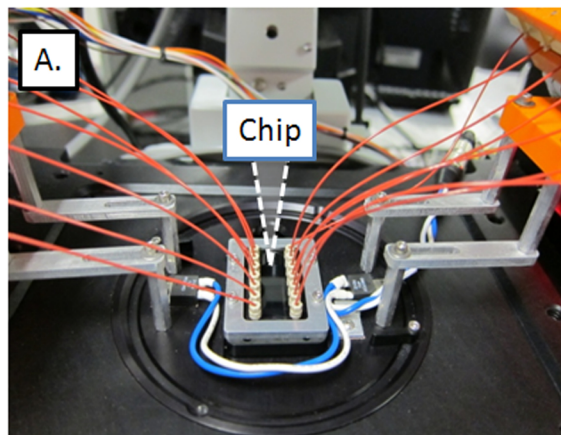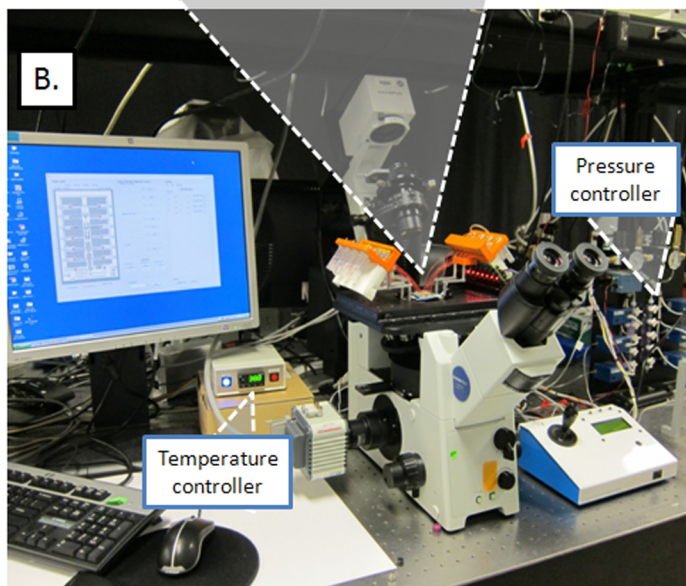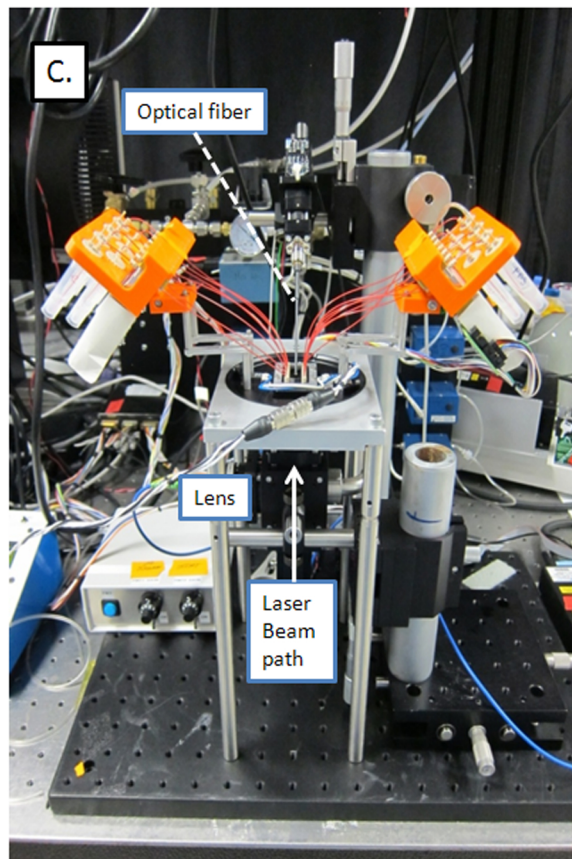

Supplement: Figure S1 — Details of the microfluidic platform. The planar microfluidic chip sits in a manifold designed in-house (A). Tubing connect valves and reagent reservoires to the inlets on the microfluid chip. B. The manifold is retrofitted to a commercial Olympus IX71 microscope. In-house designed software allows the experimenter to control the pressure, temperature, and valves by programming each step of the experiment to run automatically. After sample preparation, the manifold is moved to the micro flow cytometer setup shown in C, and on-chip flow cytometry is performed. The optical fiber is positioned on top of the chip, and aligned to the hydrodynamically focused path of the cells. The laser is applied from the bottom of the chip, and the signal from the the passing cells are recorded by the PMTs situlated underneath the chip. (PDF) [file pone.0055044.s001.pdf]

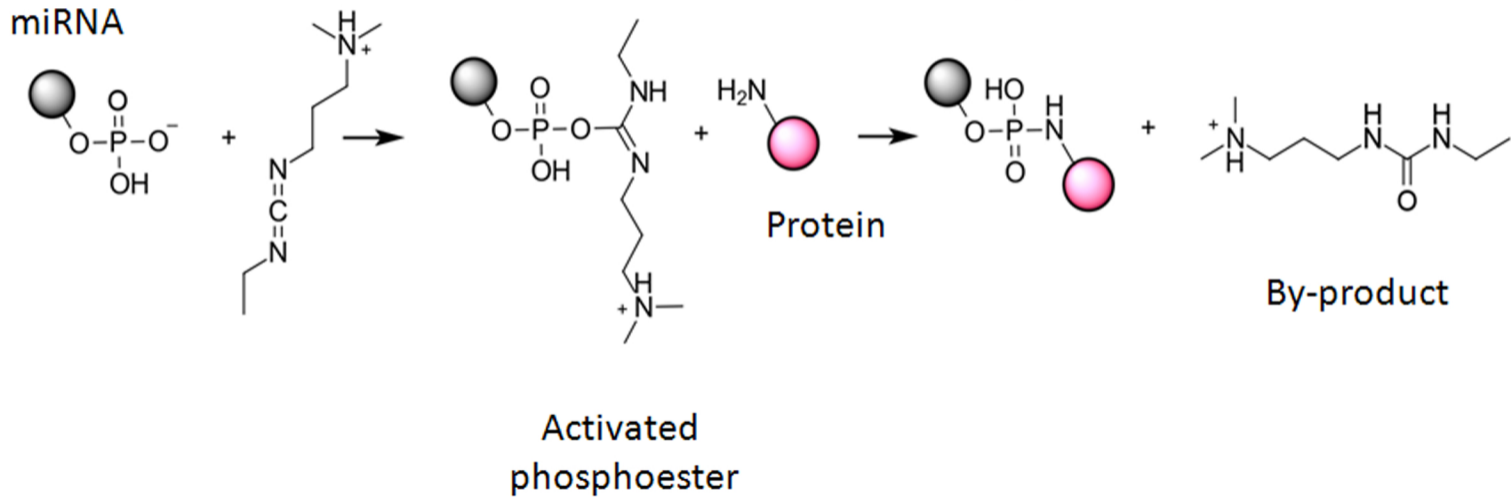

Supplement: Figure S2 — Schematic of EDC fixation mechanism. The 5′ phosphate of miRNA are activated by EDC to form an intermediate that is crosslinked to neighboring amino groups of proteins. The fixation with EDC allows irreversible crosslinking to tether miRNAs inside the cells during the high temperature hybridization with LNA containing probes. (PDF) [file pone.0055044.s002.pdf]

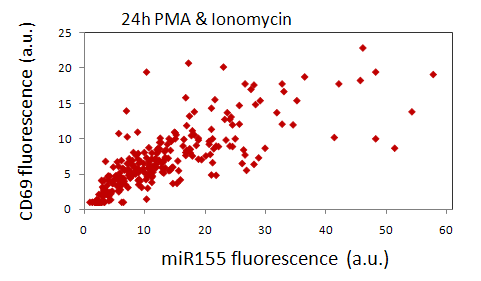

Supplement: Figure S3 — Scatter plot of Jurkat cells after 24 h of PMA and Ionomycin activation, showing heterogeneity of CD69 and miR155 expression levels in individual cells. (TIF) [file pone.0055044.s003.tif]
